# Gestational Age, Infection, and Suboptimal Maternal Prepregnancy BMI Independently Associate with Placental Histopathology in a Cohort of Pregnancies without Major Maternal Comorbidities

**DOI:** 10.3390/jcm13123378

**Published:** 2024-06-08

**Authors:** Eleanor Duffley, David Grynspan, Hailey Scott, Anthea Lafrenière, Cherley Borba Vieira de Andrade, Enrrico Bloise, Kristin L. Connor

**Affiliations:** 1 Department of Health Sciences, Carleton University, Ottawa, ON K1S 5B6, Canada; eleanorduffley@cmail.carleton.ca (E.D.); haileyscott@cmail.carleton.ca (H.S.); 2 Children’s Hospital of Eastern Ontario, Department of Pathology, Ottawa, ON K1H 8L1, Canada; david.grynspan@interiorhealth.ca; 3 Department of Pathology and Laboratory Medicine, University of British Columbia, Vancouver, BC V6T 2B5, Canada; 4 Department of Pathology and Laboratory Medicine, The Ottawa Hospital, The University of Ottawa, Ottawa, ON K1H 8L6, Canada; anthea.lafreniere@gmail.com; 5 Department of Pathology and Immunology, Baylor College of Medicine, Texas Children’s Hospital, Houston, TX 77030, USA; 6 Histology and Embryology Department, Roberto Alcantara Gomes Institute of Biology, Rio de Janeiro State University, Rio de Janeiro 20551-030, Brazil; cherleyborba@gmail.com; 7 Department of Morphology, Federal University of Minas Gerais, Belo Horizonte 31270-901, Brazil; ebloise@icb.ufmg.br; 8 Department of Physiology, University of Toronto, Toronto, ON M5S 1A8, Canada

**Keywords:** underweight, obesity, placental pathology, placental morphometry

## Abstract

**Background:** The placenta undergoes morphological and functional adaptations to adverse exposures during pregnancy. The effects ofsuboptimal maternal body mass index (BMI), preterm birth, and infection on placental histopathological phenotypes are not yet well understood, despite the association between these conditions and poor offspring outcomes. We hypothesized that suboptimal maternal prepregnancy BMI and preterm birth (with and without infection) would associate with altered placental maturity and morphometry, and that altered placental maturity would associate with poor birth outcomes. **Methods:** Clinical data and human placentae were collected from 96 pregnancies where mothers were underweight, normal weight, overweight, or obese, without other major complications. Placental histopathological characteristics were scored by an anatomical pathologist. Associations between maternal BMI, placental pathology (immaturity and hypermaturity), placental morphometry, and infant outcomes were investigated for term and preterm births with and without infection. **Results**: Fetal capillary volumetric proportion was decreased, whereas the villous stromal volumetric proportion was increased in placentae from preterm pregnancies with chorioamnionitis compared to preterm placentae without chorioamnionitis. At term and preterm, pregnancies with maternal overweight and obesity had a high percentage increase in proportion of immature placentae compared to normal weight. Placental maturity did not associate with infant birth outcomes. We observed placental hypermaturity and altered placental morphometry among preterm pregnancies with chorioamnionitis, suggestive of altered placental development, which may inform about pregnancies susceptible to preterm birth and infection. **Conclusions**: Our data increase our understanding of how common metabolic exposures and preterm birth, in the absence of other comorbidities or complications, potentially contribute to poor pregnancy outcomes and developmental programming.

## 1. Introduction

Maternal underweight and obesity are global health burdens and there has been a substantial increase in the prevalence of these conditions among women of reproductive age worldwide [1,2]. Mothers who are underweight or have obesity are at an increased risk of delivering preterm [3], which is associated with neurodevelopmental disorders and cardiometabolic diseases later in life [4,5,6]. The mechanisms that drive the relationship between suboptimal maternal body mass index (BMI) and adverse offspring outcomes in preterm and term pregnancies remain poorly understood, in part because cases are often confounded by multiple comorbidities and adverse perinatal events, making it difficult to disentangle the effects of specific exposures on fetoplacental development.

The placenta responds to cues in the pregnancy environment through morphological and functional changes in an effort to maintain proper fetal growth and development [7]. For example, delayed maturation of the placenta has been observed in response to increasing maternal BMI [7]. This altered placental maturity may result in poor gas and nutrient exchange at the maternal–fetal interface and, subsequently, suboptimal infant outcomes [8,9]. For example, delayed placental maturation, including the persistent thickness of vasculosyncytial membranes, forfeits optimal gas exchange and has been associated with placental insufficiency [10] and fetal macrosomia [11,12]. In pregnancies with gestational diabetes mellitus, placentae from pregnancies with macrosomic babies have been characterized by reduced umbilical artery Pulsatility Index compared with controls (non-macrosomic newborns). Thus, suboptimal maternal metabolic status associates with structural and functional changes in the placenta, influencing infant growth [13]. While the effects of maternal undernutrition on placental maturity are less studied, animal models of undernutrition have shown evidence of abnormal placental development, including reduced relative proportion of the junctional zone and lower cross-sectional area of fetal blood spaces [14], which may have functional consequences. The histomorphology of the placenta ultimately determines placental function, and histological markers of placental maturity and morphometry are thus clinically useful and may reveal mechanisms underlying poor offspring outcomes in the context of suboptimal maternal BMI. Yet, the limited evidence on the effect of suboptimal maternal BMI on placental maturity and morphometry stems predominantly from complicated pregnancies, and the effects of suboptimal maternal BMI alone across gestational age/infection groups on placental histomorphology remain unclear.

Independent of maternal BMI, morphofunctional changes in the placenta also occur in response to increasing maternal–fetal exchange demands throughout fetal development [15]. Compared to term placentae, preterm placentae have distinct gross and microscopic characteristics, including decreased presence of syncytial knots, thickening of the syncytiotrophoblast [16,17], and increased placental vascular lesions and evidence of malperfusion [18], pathologies that have functional consequences for the placenta. For example, accelerated maturation of the placenta has been observed in preterm pregnancies and has been interpreted as an attempted compensatory adaption [19], yet it associates with adverse fetoplacental outcomes, including relative placental insufficiency and late onset intrauterine growth restriction (IUGR) [20]. Moreover, chorioamnionitis due to infection of the fetal membranes results in an inflammatory cascade within the fetal membranes causing premature rupture ofmembranes (PROM) and preterm birth (PPROM), which can further contribute to poor fetoplacental outcomes [21]. Collectively, placental (mal)adaption in response to suboptimal maternal metabolic status, infection, and/or preterm birth may have negative effects on placental function, and thus, offspring growth and development [9]. While previous studies have associated maternal BMI with placental pathology at term, these studies do not include the full range of suboptimal maternal BMI groups, or consider preterm pregnancies with or without infection [22,23]. Others have observed placental pathological and inflammatory lesions, yet fail to exclude maternal conditions associated with inflammation and placental pathology (such as chorioamnionitis and chronic maternal inflammatory conditions) [7]. Thus, the effects of suboptimal maternal BMI on placental pathological and morphometric phenotypes in preterm (with and without chorioamnionitis) and term pregnancies without obstetric complications or comorbidities have been poorly quantified. This limits our understanding of how common metabolic exposures influence placental development at term and preterm and potentially contribute to the programming of offspring development.

To address this gap, we assessed the effects of suboptimal maternal prepregnancy BMI, without other major comorbidities, on histopathological indicators of placental maturity and morphometry in preterm (with and without chorioamnionitis) and term pregnancies. We also investigated the effect of gestational age and infection status on placental maturity and morphometry inclusive of maternal BMI, as gestational age and infection independently associate with altered placental histopathology. Lastly, we explored whether altered placental maturity was associated with suboptimal infant anthropometry and Apgar scores at birth. We hypothesized that maternal underweight (UW), overweight (OW), and obesity (OB) would associate with suboptimal placental maturity and morphometry, and that altered placental maturity would associate with poor infant birth anthropometry and Apgar scores. By characterizing the placental pathological and morphological phenotypes of pregnancies complicated by suboptimal maternal metabolic status, preterm birth, and infection, our work may uncover mechanisms that can explain poor offspring development in these pregnancies, and placental-specific histological markers that could predict altered postnatal health trajectories.

## 2. Materials and Methods

### 2.1. Study Population

This study was approved by the Mount Sinai Hospital Research Ethics Board (17-0186-E) and Carleton University Research Ethics Board (106932). Clinical data and placentae from 96 pregnancies were collected through the Research Centre for Women’s and Infants’ Health (RCWIH) BioBank at Mount Sinai Hospital, Toronto. Inclusion criteria were singleton pregnancies and live birth with no known fetal anomalies. Exclusion criteria were gestational diabetes mellitus (GDM), hypertension (including pregnancy induced hypertension), HELLP syndrome, lupus, antiphospholipid antibody syndrome, Crohn’s disease, ulcerative colitis, colitis, Guillain–Barré syndrome, sexually transmitted infections, gastritis, urinary tract infections, smokers, documented recreational drug use during pregnancy, pelvic inflammatory disease, and in vitro fertilization. Women were categorized as having delivered preterm with chorioamnionitis (PTC, n = 29), preterm without chorioamnionitis (PT, n = 31), or at term (T, n = 36; there were no term pregnancies with chorioamnionitis), and were further classified as underweight (UW, n = 21), normal weight (NW, n = 24), overweight (OW, n = 27), or as having obesity (OB, n = 24). The RCWIH Biobank established chorioamnionitis status by identifying suspected cases through signs and symptoms reported on patient charts (i.e., maternal fever, fetal tachycardia, tenderness, distinct smell of amniotic fluid during delivery), then confirming these cases with the pathologist and/or by recently obtained bloodwork. Gestational age was calculated based on the last menstrual period to the nearest week. Among 60 preterm pregnancies, 12 women were classified as UW by prepregnancy BMI, 17 as NW, 18 as OW, and 13 as OB, and among 36 term pregnancies, 9 were classified as UW, 7 NW, 9 OW, and 11 OB.

### 2.2. Maternal and Infant Characteristic Data Collection

The primary exposure of interest was maternal prepregnancy BMI classified according to the World Health Organization and American College of Obstetricians and Gynecologists guidelines [24] with one exception; due to a low prevalence of women considered underweight in the study region, a prepregnancy BMI of <19 kg/m^2^ was considered to be underweight. Maternal underweight, overweight (BMI ≥ 25–29.9 kg/m^2^), and obesity (BMI ≥ 30.0 kg/m^2^) groups were compared to normal weight controls (19–24.9 kg/m^2^). Maternal BMI was extracted directly from participant patient charts, and was considered as both a continuous and categorical exposure variable. A retrospective medical chart review was conducted to extract antenatal and birth data. Maternal characteristics have been previously reported [25]. Infant data included gestational age, infant sex, Apgar scores (at one, five, and ten minutes), and newborn anthropometry (including birthweight, a secondary outcome). Standardized birthweight by infant sex and gestational age were calculated based on singleton data reported by Kramer et al. [26].

### 2.3. Placental Collection and Processing

Placentae were collected immediately after birth by trained staff at the RCWIH BioBank (Toronto, ON, CA) by sampling a nearly full-thickness tissue core of approximately 1.5 cm by 1.5 cm by cutting from the maternal surface and excluding the chorionic plate. Placental samples were obtained from all four placental quadrants, at least 1.5 cm away from the edge and from the centre of the placental disc, the umbilical cord insertion site, and areas of thrombosis, infarcts, or other abnormalities. Biopsies were processed for histology. Formalin-fixed, paraffin-embedded placental biopsies were sectioned (6 μm) and stained with haematoxylin (Gill’s Number 1, Sigma-Aldrich, St. Louis, MO, USA) and eosin (Eosin Y-Solution, Sigma-Aldrich) and stained with (H&E) according to standard protocols. The primary outcomes of interest were placental pathologies, specifically microscopic placental pathologies related to morphometry and placental maturity. Histological chorioamnionitis was not a pre-defined histological feature evaluated here. Rather, chorioamnionitis status was determined by the BioBank from which the samples were obtained. Previously, chorioamnionitis stage and grade were assessed by a clinical pathologist in fetal membrane samples matched with this cohort [25]. Most preterm pregnancies with chorioamnionitis had fetal membranes with chorioamnionitis stage and grade 2, and all but one preterm with chorioamnionitis case had a stage and grade > 0 [25].

### 2.4. Placental Morphometry

Placental morphometry analysis was undertaken using methodology previously described in the literature with specific adaptations, which are outlined below [27]. Image acquisition and analysis were performed on a subset (n = 87) of H&E-stained sections using an Aperio AT2 microscope (Leica Biosystems, Richmond, IL, USA), coupled with a computer using the software ImageScope x64. The histomorphological analysis was undertaken by an experienced examiner, blinded to exposure groups, using the Fiji ImageJ (v1.0; ImageJ, Madison, WI, USA). Relative volume estimates of placental histological components (syncytiotrophoblast, syncytial knots, cytotrophoblasts, villous stroma [connective tissue and the villous core], and fetal capillaries) were quantified by superimposing placental histological photomicrographs with a grid of equidistant points (measuring 25 μm distance between two points). Previous studies using morphometric analysis in human placentae recorded 190–1000 points (grid intercepts) to evaluate the volumetric proportion of each placental histological component [27,28,29]. In this study, we assessed placentae by recording 1500 points overlapping with each of the histological components for the first 39 placentae. For the remaining 48 placentae, in order to optimize the recordings, we reduced the number of points recorded to 600 points overlapping with each of the histological components, while still exceeding the number of points typically assessed [29]. We analysed volumetric proportions of each histological component across BMI or gestational age groups for placentae from 1500- and 600-point morphometric analyses separately. There were no differences in volumetric proportions of histological components between the placentae assessed using the 1500-point approach and the 600-point approach when comparing outcomes across maternal BMI or gestational age groups. Thus, placentae assessed using 1500 and 600 points were pooled for statistical analyses. The total average area of evaluated histological sections per placenta was 393,173.22 μm^2^, and there were no differences in median area (μm^2^) assessed across maternal BMI groups (UW: 324,043 [273,407, 587,270]; NW: 505,332 [259,336, 557,864]; OW: 276,020 [257,202, 505,674]; OB: 502,106 [270,320, 532,097]). The volumetric proportion (VP) of each histological component was calculated as VP = NP × 100/600 for placentae for which 600 points were recorded, and as VP = NP × 100/1500 for placentae for which 1500 points were recorded, where NP = number of equivalent points on each histological component [27,30,31].

### 2.5. Placental Maturity

Histopathological characteristics were scored on H&E-stained sections by an anatomical pathologist following the Amsterdam criteria [32] to assess placental maturity and chorangiosis relative to gestational age. Hypercapillarisation and characteristics of immaturity (1. villous immaturity and 2. stromal immaturity) and hypermaturity (1. distal villous hypoplasia and 2. accelerated villous maturation) were scored as either 0 (absent) or 1 (present). Descriptions of all characteristics are included in Table 1. As previous studies in term cohorts have included accelerated villous maturation as diagnostic criteria [23,33], we assessed placental hypermaturity at both preterm and term. No placentae had both immature and hypermature characteristics.

### 2.6. Statistical Analyses

#### 2.6.1. Univariate Analyses

The primary exposure of interest was maternal prepregnancy BMI, specifically maternal UW, OW, and OB compared to NW controls. As gestational age and infection can independently affect outcomes, we also assessed these variables as secondary exposures. The primary outcomes of interest were placental maturity (immature, normal, hypermature), chorangiosis, and placental morphometry (syncytiotrophoblast, syncytial knots, cytotrophoblasts, villous stroma, and fetal capillaries). Data were stratified by term (37–42.2 weeks gestation) and preterm (<37 weeks gestation) to assess the relationships between maternal prepregnancy BMI and outcome variables in preterm and term pregnancies separately. Associations between maternal prepregnancy BMI groups or preterm with chorioamnionitis/preterm without chorioamnionitis and continuous outcomes (placental morphometry measures) were tested using one-way ANOVA or Kruskal–Wallis test with Tukey’s post hoc or Steel–Dwass post hoc. Likelihood Ratio Chi Square tests were used to evaluate the associations between maternal BMI groups or preterm with chorioamnionitis/preterm without chorioamnionitis/term group and categorical outcome variables (placental maturity outcomes). To explore sex differences in placental maturity and morphometry in response to both BMI and gestational age/infection, we also stratified data by fetal sex to analyze outcomes in males and females separately. Data were analysed using JMP statistical software (version 14.2, SAS Institute, Cary, NC, USA). Data are presented as median (interquartile range; non-parametric data), mean and standard deviation (parametric data), or frequency (percentage; categorical variables). Statistical significance was defined as *p* < 0.05. We also report false discovery rate (FDR) adjusted *p*-values (q-values).

To support the objectivity and reproducibility of our histopathological assessments, we associated our placental pathology data with morphometry histological components for which we would expect associations with placental pathology. We assessed volumetric proportion of syncytial knots or fetal vascular endothelium stratified by placental maturity (immature, normal, and hypermature) or placental hypercapillarisation using Kruskal–Wallis test with Steel–Dwass post hoc. Data are presented as median (interquartile range; non-parametric data). Statistical significance was defined as *p* < 0.05.

#### 2.6.2. Multivariable Analyses

Multivariable regression analyses were conducted to assess the relationship between maternal prepregnancy BMI (continuous) and placental maturity and morphometry separately for preterm and term pregnancies. Covariables were identified a priori. Covariables of interest were identified a priori and included fetal sex [34],maternal GWG [35,36], chorioamnionitis [37,38], and degree of prematurity (gestational age) [39]. First, an unadjusted model was used to identify the associations between prepregnancy BMI and placental maturity in preterm and term pregnancies (model A). An adjusted nominal logistic regression model was then used to determine the associations between maternal BMI and placental maturity (model B) adjusted for fetal sex (male/female), and GWG (continuous) for term pregnancies, and also adjusting for chorioamnionitis (yes/no) and gestational age (continuous) for preterm pregnancies. Odds ratios were derived from the exponential function of the regression coefficient. Data are presented as odds ratios (OR) (or adjusted OR [aOR]) and 95% confidence intervals, and *p* value from Likelihood Ratio Chi Square test. Thirdly, an unadjusted model was used to identify the associations between prepregnancy BMI and placental morphometry data (model C). An adjusted Standard Least Squares regression model was used to determine the associations between maternal BMI and placental morphometry data (model D; adjusted for the same covariates as model B. Data are presented as β (or adjusted β [aβ]) and 95% confidence intervals, and *p* value from Standard Least Squares regression models.

## 3. Results

### 3.1. Maternal BMI Has Limited Effect on Placental Maturity or Morphometry

**Figure 1:**
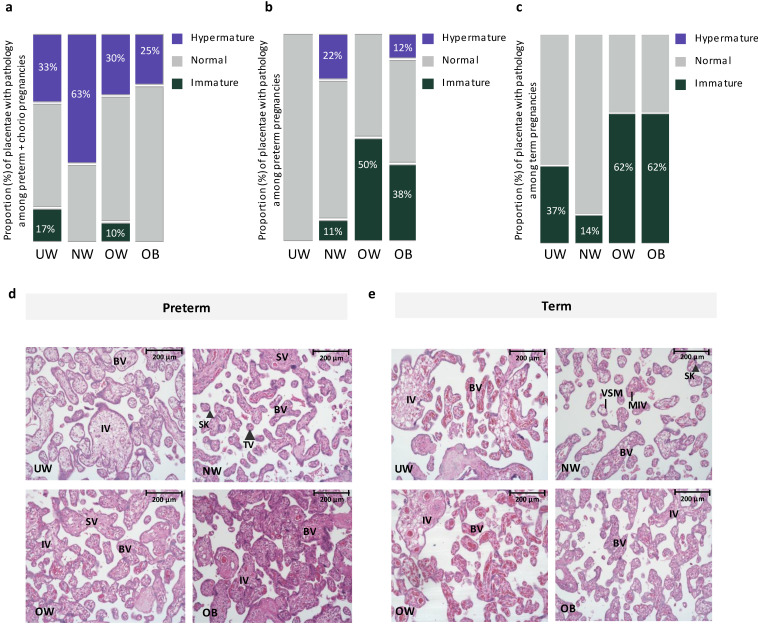
(**a**). Proportion of placentae with immature, normal, and hypermature placentae across BMI and gestational age groups among preterm pregnancies with chorioamnionitis. Placental hypermaturity was more prevalent in normal-weight pregnancies, representing a 150% and 400% increase in proportion of placentae that were hypermature in NW pregnancies, compared to UW and OW pregnancies, respectively. (**b**). Proportion of placentae with immature, normal, and hypermature placentae across BMI and gestational age groups among preterm pregnancies without chorioamnionitis. Placental immaturity was more prevalent in OW and OB pregnancies, representing a 300% and 200% increase in proportion of placentae that were immature in OW and OB, respectively, compared to NW pregnancies. (**c**). Proportion of placentae with immature, normal, and hypermature placentae across BMI and gestational age groups among term pregnancies. At term, placental immaturity was more prevalent in OW and OB pregnancies, representing a 400% increase in proportion of placentae that were immature in both OW and OB, compared to NW pregnancies. No hypermaturity was observed in term placentae. (**d**). Representative images from H&E-stained placentae from UW, NW, OW and OB preterm pregnancies. UW, OW, OB = immature pathology. BV = Blood vessel, IV = immature villus, TV (large arrowhead) = terminal villus, VSM = Vasculo-syncytial membrane, SK (small arrowhead) = Syncytial knot, SV = Stem villus. 20× Magnification. Scale bar = 200 μm. (**e**). Representative images from H&E-stained placentae from UW, NW, OW and OB term pregnancies. UW, OW, OB = immature pathology. BV = Blood vessel, IV = immature villus, MIV = mature intermediate villus, VSM = Vasculo-syncytial membrane, SK (large arrowhead) = Syncytial knot. 20× Magnification. Scale bar = 200 μm.

There was no effect of maternal prepregnancy BMI on placental anthropometry among preterm or term pregnancies (Table 2). However, based on histopathologic classification by an anatomic pathologist, we found that, among preterm pregnancies with chorioamnionitis, placental hypermaturity was more prevalent in NW pregnancies compared to UW and OW pregnancies, where UW and OW pregnancies had a 150% and 400% decrease in proportion of hypermature placentae, respectively, compared to NW pregnancies (Figure 1). At preterm (without chorioamnionitis), placental hypermaturity was not highly prevalent with suboptimal BMI, but immaturity was more prevalent in OW and OB preterm pregnancies compared to NW pregnancies, representing a 300% and 200% increase in the proportion of immature placentae, respectively (Figure 1). At term, placental immaturity was more prevalent in OW and OB pregnancies, representing a 400% increase in proportion of placentae that were immature in OW and OB pregnancies, respectively, compared to NW pregnancies. However, there were no differences in placental maturity (immature, normal, hypermature) across maternal BMI groups when stratifying by preterm with chorioamnionitis, preterm without chorioamnionitis, and term pregnancies (Figure 1). Among preterm pregnancies, when considering BMI as a continuous variable, odds of placental immaturity ([model A: OR = 1.06 (*−*0.03, 0.16), *p* = 0.28]; [model B: aOR = 1.09 (*−*0.03, 0.21), *p* = 0.21]) and odds of placental hypermaturity ([model A: OR = 0.97 (*−*0.15, 0.07), *p* = 0.56]; [model B: aOR = 1.01 (*−*0.11, 0.13), *p* = 0.83]) did not change with each one unit increase in maternal BMI. Odds of placental immaturity also did not change with each one unit increase in maternal BMI among term pregnancies ([model A: OR = 1.05, (0.95, 1.16), *p* = 0.27]; [model B: aOR = 1.07 (0.95, 1.19), *p* = 0.24]). When data were further stratified by fetal sex, there were no differences in placental maturity across maternal BMI groups at preterm and term in male or female placentae (Supplementary Tables S1 and S2). At term, hypercapillarisation was more prevalent in OW (5 [62.5]) and OB (3 [37.5]) pregnancies, compared to NW (0 [0.0]) and UW (1 [12.5]) pregnancies (*p* = 0.01). Hypercapillarisation was not present in preterm pregnancies or preterm pregnancies with chorioamnionitis.

**Figure 2:**
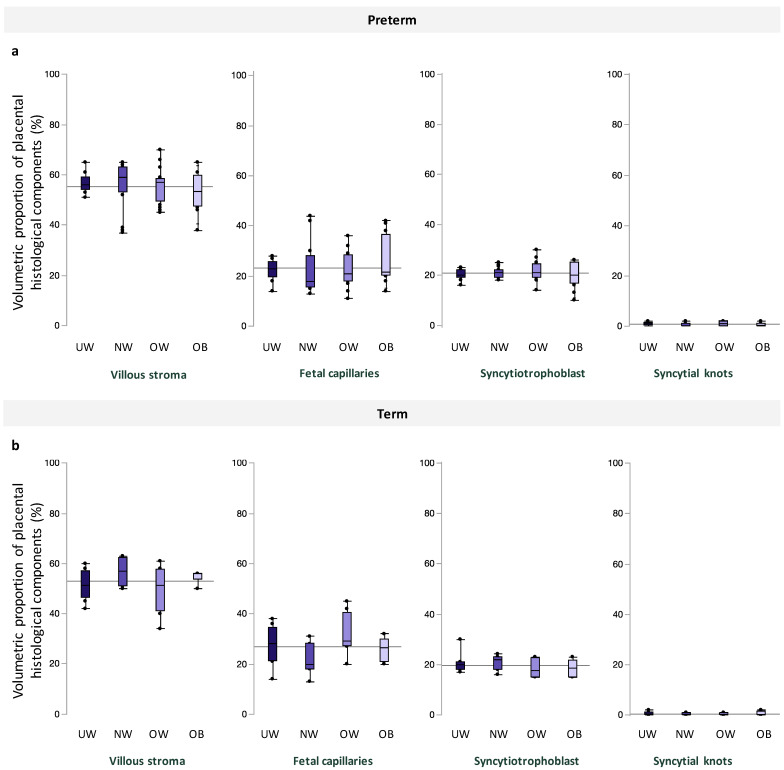
Volumetric proportion of placental histological components (%), including, from left to right, villous stroma, fetal capillaries, syncytiotrophoblast, and syncytial knots across maternal BMI groups among (**a**). preterm and (**b**). term pregnancies. Data are quantile box plots with a horizontal line representing the mean across the whole cohort.

Among preterm and term pregnancies, villous stroma comprised the greatest area of quantified tissue, followed by fetal capillaries, syncytiotrophoblast, and syncytial knots, and there were no differences in volumetric proportion of these histologic features across maternal BMI groups (Figure 2). Placental morphometry also did not differ with increasing maternal BMI (Table 3). When data were stratified by fetal sex, there was no association between maternal BMI and volumetric proportion of any histological components at preterm and term in male or female placentae (Supplementary Tables S1 and S2).

### 3.2. Maternal BMI Has Limited Effect on Birth Outcomes

At birth, standardized birthweight increased with increasing maternal BMI among preterm and term infants (Table 2); although, among term infants there were no differences in infant birthweight z-scores (BWZ) between BMI groups on post hoc analysis. There were no differences across maternal BMI groups for infant Apgar scores at 1 or 5 min among preterm or term infants (Table 2).

### 3.3. Gestational Age and Infection Status Associate with Altered Placental Maturity and Morphometry

We found that placental weight (*p* < 0.0001) and birthweight-to-placental weight ratio (*p* < 0.0001), sometimes used as a proxy measure for placental efficiency (as well as placental developmental stage), were decreased in preterm pregnancies with chorioamnionitis compared to preterm without chorioamnionitis and term pregnancies, inclusive of BMI (Table 4). As expected, gestational age was also lower in preterm with chorioamnionitis compared to preterm pregnancies without chorioamnionitis and term pregnancies (Table 4). Additionally, inclusive of BMI, the greatest proportion of placental hypermaturity was observed in preterm pregnancies with chorioamnionitis [immature = 1 (3.7), normal = 15 (55.6), hypermature = 11 (40.7)] compared to preterm [immature = 8 (27.6), normal = 18 (62.1)], hypermature = 3 (10.3)] and term [immature = 14 (45.2), normal = 17 (54.8), hypermature = 0 (0.00)] pregnancies (*p* < 0.0001). In further exploring this association, we found that, while preterm pregnancies with chorioamnionitis had increased odds of accelerated villous maturation (AVM), but not distal villous hypoplasia (DVH), compared to preterm pregnancies without chorioamnionitis (*p* = 0.01), the significance of this difference was not retained after adjusted analyses (Supplementary Table S5). Prevalence of immaturity, normal maturity, and hypermaturity did not differ across gestational age/infection groups when stratified by fetal sex (Supplementary Table S3).

Fetal capillary volumetric proportion was decreased (*p* = 0.05, q = 0.13, Table 5) and villous stromal volumetric proportion was increased (*p* = 0.02, q = 0.1, Table 5) in preterm pregnancies with chorioamnionitis compared to preterm pregnancies without chorioamnionitis. Although, there were no differences in fetal capillary volumetric proportion on post hoc analysis, and there were no differences in fetal capillary volumetric proportion or villous stromal volumetric proportion following FDR adjustment. Infection status at preterm had no effect on syncytial knots, syncytiotrophoblast, or cytotrophoblast volumetric proportions (Table 5). When data were stratified by fetal sex, there were no differences in volumetric proportion of histologic features in male or female placentae in preterm pregnancies with chorioamnionitis compared to preterm pregnancies without chorioamnionitis (Supplementary Table S4).

### 3.4. Preterm Pregnancies with Chorioamnionitis Associate with Decreased Infant Apgar Scores

**Figure 3:**
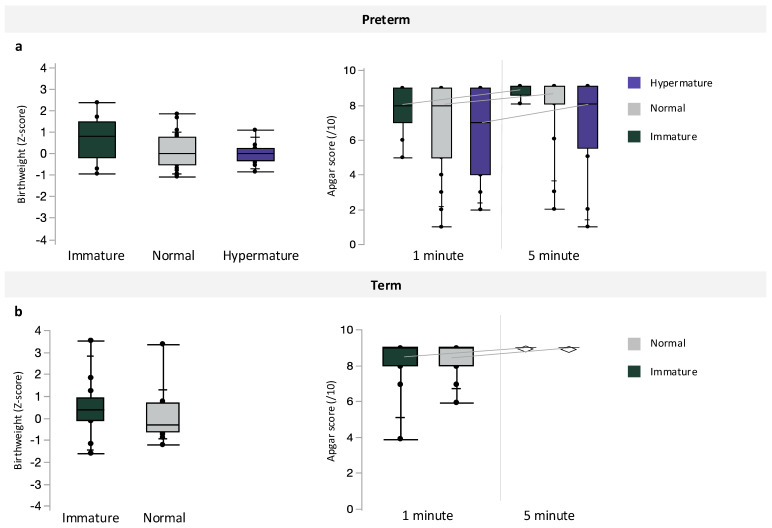
Associations between placental maturity (immature, normal, and hypermature) and birthweight z-scores or Apgar scores at 1 and 5 min among (**a**) preterm and (**b**) term pregnancies. Data are quantile box plots.

Inclusive of maternal BMI, preterm pregnancies with chorioamnionitis had the lowest median infant birthweight, followed by preterm pregnancies without infection and term pregnancies (*p* < 0.0001, Table 4). However, there were no differences in infant BWZ between preterm, preterm with chorioamnionitis, and term pregnancies (Table 4). Apgar scores at one minute (*p* = 0.0003) were decreased in preterm pregnancies with chorioamnionitis, compared to scores in term infants, and Apgar scores at 5 min were also decreased in both preterm pregnancies with and without chorioamnionitis compared to term infants (*p* = 0.0003, Table 4). Preterm pregnancies with chorioamnionitis also had the lowest median gestational age at birth, followed by preterm pregnancies without infection and term pregnancies (*p* < 0.0001, Table 4). Placental maturity did not associate with infant BWZ or Apgar scores at 1 and 5 min (Figure 3) among preterm and term pregnancies.

### 3.5. Placental Maturity and Chorangiosis Associate with Placental Morphometry

**Figure 4:**
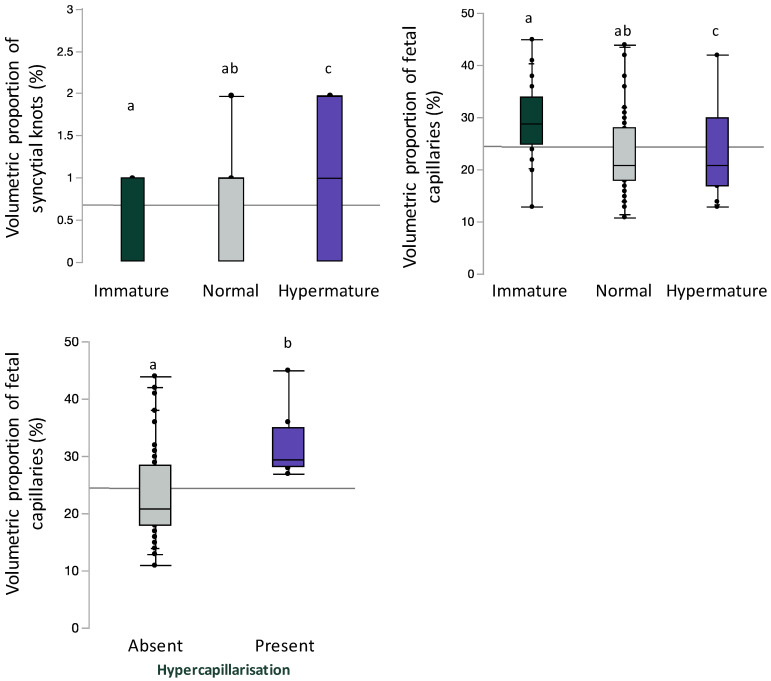
Volumetric proportion of syncytial knots or fetal vascular endothelium stratified by placental maturity (immature, normal, and hypermature) or placental hypercapillarisation. Data are quantile box plots with a horizontal line representing the mean across the whole cohort. Differences between groups are denoted by different lowercase letters.

Inclusive of BMI and gestational age, syncytial knot volumetric proportion was increased (immature 0 [0, 1]; normal 1 [0, 1], hypermature 1 [0, 2]; *p* = 0.04) and fetal capillary volumetric proportion was decreased (immature 29 [25, 34]; normal 21 [18, 28]; hypermature 21 [17, 30]; *p* = 0.003) with advancing maturity; significant differences were only observed between hypermature placentae compared to immature placentae on post hoc analysis (Figure 4). Inclusive of maternal BMI and gestational age, fetal capillary volumetric proportion was increased in placentae with hypercapillarisation compared to placentae without hypercapillarisation (absent 21 [18, 28.5]; present 29.5 [28.3, 35], *p* = 0.003, Figure 4).

## 4. Discussion

We examined the effect of maternal prepregnancy BMI, without other major comorbidities, on placental maturity and morphometry to quantify how suboptimal maternal metabolic states influence placental phenotypes and to better understand the mechanisms that may contribute to poor pregnancy outcomes and fetal (mal)development in these pregnancies. Reassuringly, we found no major differences in placental maturity or morphometry across maternal prepregnancy BMI groups among preterm or term pregnancies and placental maturity did not associate with infant birthweight or Apgar scores at birth. There were limited associations between maternal BMI and infant birth outcomes. We did observe an influence of gestational age and infection on placental phenotypes, where the greatest proportion of hypermature placentae were from preterm pregnancies with chorioamnionitis, compared to placentae from preterm pregnancies without infection and term pregnancies. Accordingly, preterm pregnancies with chorioamnionitis were associated with decreased placental weight and efficiency and decreased infant Apgar scores, suggesting that infection in the context of preterm birth may have negative implications for placental development and infant outcomes.

Our data showed limited evidence for an effect of low or high prepregnancy BMI on placental maturity and morphometry among preterm or term pregnancies. Among both term and preterm pregnancies, we found that placental immaturity was more prevalent in OW and OB pregnancies, representing a high percentage increase in the proportion of placentae that were immature in both OW and OB pregnancies, compared to NW pregnancies, which may suggest the emergence of underlying pathology. We observed hypercapillarisation only at term, where it was more prevalent in term OW and OB pregnancies compared to NW and UW pregnancies. In our study, placentae from pregnancies of mothers who were overweight or had obesity displayed a phenotype similar to placentae from pregnancies complicated by type 1 diabetes, which are generally larger than normal, immature, and hypercapillarised [12]. It is well established that maternal obesity promotes a pro-inflammatory environment within gestational tissues, including elevated levels of circulating interleukin IL-6 during pregnancy and higher levels of placental pro-inflammatory cytokines [40], and associates with poor infant outcomes. Further, a recent study observed that placentae from pregnancies with GDM and increased prepregnancy BMI or GWG had increased expression of neoangiogenesis and inflammatory markers, such as vascular endothelial growth factor (VEGF) and CD31 [35]. Diet-induced maternal obesity also associates with increased levels of the proinflammatory cytokines tumor necrosis factor TNF-α and IL-8 in sheep placentae [40]. TNF-α can inhibit placental trophoblast motility and migration, indicating its potential to impact placental development [41]. Thus, poor maternal metabolic health is permissive of a pro-inflammatory environment that may adversely affect normal placental maturity and structure. However, in contrast to studies showing increased proportion of macroscopic and microscopic placental pathologies with increasing maternal BMI [7,42], our data show limited evidence for altered placental histopathology in pregnancies with suboptimal maternal BMI. Differences in our findings could be explained by our study design. We intentionally excluded pregnancies with comorbidities and complications that are associated with obesity and underweight and have known effects on placental development and function [12,43] so that we could more accurately gain insight into the effects of suboptimal BMI alone on placental pathology and morphometry. Hypertension and GDM are highly prevalent in mothers who are overweight or have obesity [44] and could be driving the placental histopathological changes previously reported in pregnancies complicated by suboptimal maternal BMI. Given that an estimated 30% of women with overweight or obesity have no other comorbidities [45,46], our limited histomorphological findings in placentae from otherwise uncomplicated pregnancies may be reassuring.

Gestational age and infection may also alter placental pathology and morphometry. Indeed, our placental morphometric analyses show a modest decrease in fetal capillary volumetric proportion in preterm pregnancies with chorioamnionitis compared to preterm pregnancies without chorioamnionitis. Likely due to this global reduction in vascularity, we also observed increased villous stromal volumetric proportion in preterm pregnancies with chorioamnionitis compared to preterm pregnancies without chorioamnionitis. Endothelial cell proliferation and elongation is critical for placental vascular remodeling throughout pregnancy [47], and placental endothelial cell dysfunction can contribute to the development of disorders such as placental insufficiency and pre-eclampsia [48]. Thus, decreased fetal capillary volume fraction in preterm pregnancies with chorioamnionitis may suggest inadequate placental vasculature and decreased placental blood flow throughout gestation, and possible associations between gestational age and infection with placental morphometry need to be explored in other and larger cohorts to determine if they can be replicated elsewhere. 

In what may have been an attempted compensatory adaptation to decreased fetal capillaries, placental blood flow, and subsequent placental hypoxia [49], preterm pregnancies with chorioamnionitis also showed a greater proportion of placental hypermaturity compared to preterm and term pregnancies. While we observed placental hypermaturity in both preterm pregnancies with and without chorioamnionitis, the observation of greater hypermaturity in PTC compared to PT is unlike previous studies [49]. In further exploring this association, we found that preterm with chorioamnionitis pregnancies had increased odds of AVM, but not DVH, compared to preterm pregnancies without chorioamnionitis, though there were no differences in odds of AVM or DVH in preterm pregnancies with chorioamnionitis compared to preterm pregnancies without chorioamnionitis after adjusting for fetal sex, maternal GWG, and gestational age. Because our exclusion criteria precluded most cases with pre-placental major maternal comorbidities and maternal conditions associated with placental underperfusion, we were left with a cohort that was likely all “spontaneous”; that is, etiologies that included threatened preterm labor, chorioamnionitis, preterm premature rupture of the membranes, and cervical incompetence. However, there may be some indicated preterm births in our cohort, which may be represented to a greater degree at later gestational ages and may thus influence the placental outcomes we measured here or could explain the lack of chorioamnionitis we see in these later ages. Our results may raise the possibility that those with histological chorioamnionitis may be distinct from the rest of the spontaneous (non-iatrogenic) preterm cluster by having, perhaps, long-standing adaptation via accelerated maturation to (occult) placental insufficiency, perhaps due to a global reduction in fetal vasculature (proposed mechanism depicted in Supplementary Figure 2). This is in line with previous findings of placental molecular changes in cases of chorioamnionitis. Indeed, others have shown that placental fetal capillaries are stressed by chorioamnionitis, independent of gestational age; angiogenic factors were decreased, and factors linked to microvessel maturation were increased in placentae from pregnancies with chorioamnionitis compared to gestational age-matched controls without placental inflammation [38]. Thus, our work may support the hypothesis that chorioamnionitis impairs fetal capillary angiogenesis, and as a result, may lead to placental hypermaturation as an attempted compensation. However, as PTC pregnancies delivered at earlier gestational age compared to the PT and T groups, differences in developmental stage (related to gestational age) and possible secondary villous edema, which was not assessed here but has been associated with chorioamnionitis, may also play a role. While we separated term and preterm pregnancies, our findings remain to be fully explored in future, larger cohorts to disentangle the effects of gestational age and chorioamnionitis on placental histopathology.

To corroborate our placental pathology data, we compared immature, normal, and hypermature placentae and found that syncytial knot volumetric proportion was increased and fetal capillary volumetric proportion was decreased in hypermature placentae compared to immature placentae. Increased syncytial knots in hypermature placentae is to be expected, as this is a hallmark of placental maturation. However, the decline in fetal vascular volume fraction with maturity has not yet been studied. Previous studies using stereology assessed fetal vessels, such as Mayhew and colleagues who used fetal vascular length and surface area calculations [50]. One potential explanation is that along with the maturational changes in villous shape there is a maturational change in villous stromal and vascular design. The central vessels—in stem villi—become more muscularised and distributive, and the terminal villi develop less central vascularisation and acquire vasculosyncytial membranes. Our finding that this results in a global reduction in volume fraction is novel, and merits further investigation. We also found that fetal capillary volumetric proportion was increased in placentae with hypercapillarisation compared to placentae without hypercapillarisation. Given that our morphometric analysis of fetal capillary volumetric proportion quantified all points falling on villous capillaries, our finding of greater fetal capillary volumetric proportion in placentae with increased number of capillaries (chorangiosis) thus corroborates this pathology assessment. Taken together, these data support the validity of our findings and represent an additional approach for corroborating histological assessment.

Optimal placental function is necessary for the delivery of nutrients, oxygen, and hormones to the developing fetus [51].While we observed no differences in placental maturity in preterm birth without infection compared to term pregnancies, we found decreased placental weight and efficiency in preterm pregnancies without infection compared to term pregnancies. Others have suggested that placental insufficiency, including various placental pathologies such as placental hypermaturity and reduced placental weight [52], is one etiology of idiopathic preterm birth which may arise from oxidative stress due to abnormal spiral artery remodeling and subsequent suboptimal uteroplacental blood flow [52,53]. The histological markers of placental immaturity and hypermaturity are also indicative of a placenta that may be structurally ill-suited to meet fetal demands [11,12,20]. However, in our cohort, placental maturity did not associate with infant outcomes at birth in preterm or term pregnancies. This is surprising, as others have supported the prognostic value of placental histology, including demonstrating associations between placental maturity and infant outcomes [49,54]. Whereas previous studies linking placental maturity and infant birth outcomes were from complicated pregnancies, our cohort purposefully lacked major maternal comorbidities apart from suboptimal maternal BMI. This suggests that altered placental maturity may only predict infant outcomes in complex pregnancies with specific comorbidities. Altered placental pathology has also been associated with long-term adverse offspring outcomes. For example, others have demonstrated associations of villous edema, maternal vascular malperfusion, and funisitis in preterm-born pregnancies with suboptimal offspring neurodevelopmental outcomes at school age [55,56]. Also, conditions that alter placental pathology associate with long-term adverse maternal phenotypes [57]. For example, hypertensive disorders of pregnancy associate with defective spiral artery remodeling and later maternal cardiovascular disease [58,59]. Long-term follow up of mother–infant dyads with placental pathology is required to determine whether there are long-term adverse offspring and maternal outcomes in pregnancies without major complications or maternal comorbidities. A key strength of our study is the exclusion of pregnancies with major comorbidities and conditions that may associate with pathological placental findings, including GDM, hypertension, pre-eclampsia, pro-inflammatory conditions, and in vitro fertilization. Here, we assessed placental morphometry and maturity among preterm (with and without chorioamnionitis) and term pregnancies to better understand the influence of the full range of suboptimal maternal BMI at these gestational periods on placental development. To our knowledge, only one other study has evaluated placental histopathology in pregnancies with obesity without complications or comorbidities, and this study reported only moderate associations between increasing maternal BMI and accelerated villous maturation and chronic villitis among term pregnancies [23]. In contrast, a study by Bar et al. investigating high prepregnancy BMI with maternal conditions including pre-eclampsia and GDM, but not hypertension or other pro-inflammatory conditions [22], showed increased maternal inflammatory lesions among pregnancies complicated by obesity compared to normal-weight pregnancies; these findings were consistent when comparing mothers with and without complications [22]. However, this cohort did not assess underweight or preterm pregnancies, and as such, did not capture the full scope of metabolic states or gestational age effects as we did. Another larger study by Huang et al. that included a cohort of women with pregnancy complications observed increased placental pathology with increasing maternal BMI, a finding that was also observed in a subset of women without obstetric complications; however, preterm and term pregnancies were not examined separately [7]. Thus, there are conflicting findings and discrepancies in cohort selection among the few studies investigating the effects of suboptimal maternal BMI on placental pathology. While future studies are required to confirm the effects of suboptimal maternal BMI alone on placental pathology, our cohort helps to address these gaps in knowledge on the impact of maternal prepregnancy BMI, preterm birth, and infection, in the absence of other major maternal comorbidities, on placental maturity and morphometry.

## 5. Conclusions

Our data show that gestational age and infection associate with altered placental maturity and morphometry, and, at term, placental immaturity and hypercapillarisation are more likely in pregnancies with high maternal prepregnancy BMI, despite suboptimal BMI (in the absence of other comorbidities) having few other effects on placental histopathologies. Limited changes in micro/macroscopic placental pathology do not preclude functional changes in placentae from pregnancies complicated by suboptimal maternal BMI. Our results add to the incomplete evidence on the effects of suboptimal maternal BMI, gestational age, and infection on placental maturity and morphometry in pregnancies without major comorbidities, and are a step forward in understanding the mechanisms that may contribute to poor offspring outcomes in pregnancies complicated by suboptimal maternal BMI and preterm birth (with and without infection).

## Figures and Tables

**Table 1:** Methodology of placental histopathological assessment.

Histopathological Characteristic	Definition	Scoring
**Immature**	Villous immaturity [monotonous villi (≥10 villi) with centrally placed capillaries] Stromal immaturity [decreased vasculosyncytial membranes resembling villi in early pregnancy present in at least 30% of full thickness section]	[0 (absent) or 1 (present)]
**Hypermature**	Distal villous hypoplasia [reduced size of intermediate villi with dispersed terminal villi and reduced number that appear thin and elongated, widening of intervillous space; adjusted for gestational age; involving at least 30% of a full thickness slide] Accelerated villous maturation [the presence of term-appearing and/or hypermature villi for gestational age, not in areas adjacent to infarction].	[0 (absent) or 1 (present)]
**Hypercapillarisation**	Chorangiosis defined as >10 terminal villi with ≥10 capillaries.	[0 (absent) or 1 (present)]

Placentae with presence of one or more immaturity characteristic but absent of all hypermaturity characteristics were deemed ‘immature’ for gestational age. Placentae with presence of one or more hypermaturity characteristic but absent all immaturity characteristics were deemed ‘hypermature’ for gestational age. Placentae absent all immaturity and hypermaturity characteristics were deemed ‘normal’ for gestational age.

**Table 2:** Infant characteristics by prepregnancy BMI in preterm and term pregnancies.

	Prepregnancy BMI				
	UW	NW	OW	OB	*p* Value
**Infant characteristics**					
**Preterm pregnancies**	(n = 12)	(n = 17)	(n = 18)	(n = 13)	
Sex (n [%])					0.47
Female	5 (41.7)	5 (29.4)	10 (55.6)	5 (38.5)	
Male	7 (58.3)	12 (70.59)	8 (44.4)	8 (61.5)	
Z-birthweight	−0.34 ± 0.94 ^A^	−0.02 ± 0.71 ^AB^	0.20 ± 0.65 ^AB^	0.59 ± 1.09 ^B^	0.05
Birthweight (g)	1735 (1010, 2118)	1820 (1005, 2176)	1700 (960, 2685)	1820 (1510, 2955)	0.53
Placental weight (g)	410 (350, 490)	400 (330, 530)	448 (323, 690)	480 (388, 670)	0.21
Birthweight (g): placental weight (g)	4.39 ± 1.19	3.96 ± 1.23	3.73 ± 1.19	3.90 ± 1.00	0.52
Apgar score					
1 min	8 (6.25, 8)	8 (4.5, 9)	8 (5, 9)	8.5 (3.75, 9)	0.93
5 min	8.5 (8, 9)	9 (8, 9)	9 (8, 9)	9 (8, 9)	0.87
10 min ^a^	8 (8, 8)	8 (2.75, 8.75)	7 (7, 9)	9 (5, 9)	0.90
Gestational age (weeks)	32.1 (29, 34.2)	31.3 (27, 34.7)	31 (27.5, 34.8)	33.3 (29.6, 35.2)	0.81
**Term pregnancies**	(n = 9)	(n = 8)	(n = 8)	(n = 11)	
Sex (n [%])					0.05
Female	3 (33.3)	2 (37.5)	6 (66.7)	9 (81.8)	
Male	6 (66.7)	5 (62.5)	3 (33.3)	2 (18.2)	
Z-birthweight	−0.31 (−0.78, 0.11) ^A^	0.02 (−0.49, 0.75) ^A^	0.14 (−0.27, 0.66) ^A^	0.62 (0.09, 2.28) ^A^	0.04
Birthweight (g)	3280 (3075, 3480)	3500 (3230, 3820)	3630 (3160, 3780)	3550 (3310, 3940)	0.13
Placental weight (g)	500 (465, 620)	700 (563, 743)	610 (550, 780)	618 (570, 1000)	0.09
Birthweight (g): placental weight (g)	6.11 ± 0.87	5.45 ± 1.00	5.46 ± 0.74	5.15 ± 1.35	0.25
Apgar score (n [%])					
1 min	9 (8, 9)	9 (9, 9)	9 (8.5, 9)	9 (6.75, 9)	0.47
5 min	9 (9, 9)	9 (9, 9)	9 (9, 9)	9 (8.75, 9)	0.37
10 min ^a^	-	-	9	9 (9, 9)	1.00
Gestational age (weeks)	39.3 ± 0.52	39.3 ± 1.05	39.8 ± 1.30	38.7 ± 0.88	0.12

Data are means ± SD (ANOVA; normal distribution/equal variance) or median (IQR; Kruskal Wallis/Wilcoxon test for non-parametric data with Steel-Dwass post hoc). Data are n (%) (Likelihood Ratio Chi Square test) for categorical variables. Post hoc differences between groups are denoted by different letters. *p* < 0.05. Placental weights reported are from fresh, untrimmed placentae. ^a^ 10 min Apgar scores were not available for 9 (75%) preterm UW, 13 (76.5%) preterm NW, 15 (83.3%) preterm OW, and 10 (76.9%) preterm OB pregnancies. No 10 min Apgar scores were obtained for term UW or term NW groups, and scores were not available for 7 (87.5%) term OW and 9 (81.8%) term OB pregnancies.

**Table 3:** Associations between prepregnancy BMI and placental morphometry volumetric proportions in preterm and term pregnancies.

	Model C		Model D	
Volumetric Proportion of Placental Histological Components (%)	β (95% CI)	*p*-Value	aβ (95% CI)	*p* Value
**Preterm (n = 56)**				
Syncytiotrophoblast	−0.01 (−0.15, 0.12)	0.80	−0.05 (−0.19, 0.08)	0.42
Cytotrophoblast	0.001 (−0.006, 0.008)	0.74	−0.002 (−0.008, 0.004)	0.53
Villous stroma	−0.19 (−0.48, 0.09)	0.18	−0.22 (−0.53, 0.08)	0.16
Fetal Capillaries	0.21 (−0.11, 0.54)	0.19	0.28 (−0.05, 0.63)	0.09
Syncytial knots	−0.008 (−0.04, 0.02)	0.60	−0.01 (−0.04, 0.02)	0.52
**Term (n = 31)**				
Syncytiotrophoblast	−0.11 (−0.29, 0.06)	0.18	−0.08 (−0.31, 0.1)	0.42
Cytotrophoblast	−0.006 (−0.02, 0.009)	0.42	−0.002 (−0.02, 0.02)	0.82
Villous stroma	0.03 (−0.31, 0.38)	0.81	−0.07 (−0.52, 0.38)	0.73
Fetal Capillaries	0.06 (−0.32, 0.45)	0.74	0.14 (−0.37, 0.66)	0.58
Syncytial knots	−0.005 (−0.03, 0.02)	0.74	−0.002 (−0.04, 0.03)	0.63

Data are β and 95% CI and *p* value from Standard Least Squares models. *p* < 0.05. For preterm and term, model C is unadjusted. Model D adjusted for fetal sex, maternal gestational weight gain, chorioamnionitis status, and gestational age. For term, model D includes fetal sex and maternal gestational weight gain.

**Table 4:** Infant characteristics in preterm pregnancies with chorioamnionitis, preterm pregnancies without chorioamnionitis, and term pregnancies.

	Gestational Age and Infection Status			
	Preterm Chorio (n = 29)	Preterm (n = 31)	Term (n = 36)	*p* Value
Infant characteristics				
Sex (n [%])				0.37
Female	13 (44.8)	12 (38.7)	20 (55.6)	
Male	16 (55.7)	19 (61.3)	16 (44.4)	
Z-birthweight	0.02 (−0.34, 0.33)	0.3 (−0.71, 0.89)	0.14 (−0.49, 0.74)	0.85
Birthweight (g)	1130 (950, 1700) ^A^	2340 (1810, 2710) ^B^	3485 (3230, 3672) ^C^	<0.0001
Placental weight (g)	368 (303, 463) ^A^	500 (400, 650) ^B^	600 (543, 743) ^C^	<0.0001
Birthweight (g): placental weight (g)	3.43 ± 1.04 ^A^	4.43 ± 1.05 ^B^	5.54 ± 1.05 ^C^	<0.0001
Apgar score				
1 min	8 (4, 8) ^A^	8.5 (6, 9) ^AB^	9 (8, 9) ^B^	0.0003
5 min	8 (6, 9) ^A^	9 (8, 9) ^A^	9 (9, 9) ^B^	0.0001
10 min ^a^	7 (5, 8) ^A^	9 (8, 9) ^B^	9 (9, 9) ^B^	0.0048
Gestational age (weeks)	28.1 (26.6, 31.4) ^A^	34.7 (31.9, 35.6) ^B^	39.1 (38.6, 39.7) ^C^	<0.0001

Data are means ± SD (ANOVA; normal distribution/equal variance with Tukey post hoc) or median (IQR; Kruskal-Wallis/Wilcoxon test for non-parametric data with Steel-Dwass post hoc). Data are n (%) (Likelihood Ratio Chi Square test) for categorical variables. Post hoc differences between groups are denoted by different letters. *p* < 0.05. ^a^ 10 min Apgar scores were not available for 22 (75.9%) preterm chorio, 25 (80.6%) preterm, and 33 (91.7%) term pregnancies.

**Table 5:** Effect of gestational age at birth and infection status on volumetric proportion of placental histological components.

Volumetric Proportion of Placental Histological Components (%)		Gestational Age at Birth			
	Preterm with Chorioamnionitis (n = 27)	Preterm (n = 29)	Term (n = 31)	† *p* Value	† q Value
**Syncytiotrophoblast**	20 (19, 22)	21 (19, 23.2)	20 (16.5, 22.5)	0.72	0.9
**Cytotrophoblast**	0 (0, 0)	0 (0, 0)	0 (0, 0)	0.97	0.97
**Villous stroma**	58 (54, 63)	55 (48, 58.6)	55 (50, 57.5)	0.02	0.1
**Fetal capillaries**	20 (17, 26)	22 (18, 29.5)	28 (20.5, 30.5)	0.05	0.13
**Syncytial knots**	1 (0, 2)	1 (0,1)	0 (0, 1)	0.15	0.25

Data are volumetric proportions of placental histological components. Data are presented as median (IQR). **†** Comparisons are calculated only for PTC vs. PT given the impact of gestational age on development/differentiation of histological components (Kruskal–Wallis test for non-parametric data) *p* < 0.05.

## Data Availability

Data are available from the authors upon reasonable request and with permission of the Research Centre for Women’s and Infants’ Health BioBank where accessibility restrictions may apply due to the terms contained within the biobank’s material transfer agreements. Requests for data from this study can be made to Kristin Connor.

## Supplementary Materials

The following supporting information can be downloaded at: https://www.mdpi.com/article/10.3390/jcm13123378/s1, Supplementary Figure S1: Cohort breakdown; Supplementary Figure S2: Proposed mechanism of placental villous hypermaturity in preterm pregnancies with chorioamnionitis. Decreased fetal vascular endothelium and increased villous stroma, and subsequently inadequate placental vasculature and blood flow, in preterm pregnancies with chorioamnionitis may prompt compensatory placental hypermaturation. Syncytiotrophoblast shedding via increased number of syncytial knots and subsequent thinning of the syncytiotrophoblast layer may be one mechanism leading to placental villous hypermaturity and attempted improved exchange in preterm pregnancies with chorioamnionitis; Supplementary Table S1: Effect of maternal BMI group on volumetric proportions of placental histological components and placental maturity stratified by fetal sex in preterm pregnancies; Supplementary Table S2. Effect of maternal BMI group on placental maturity, hypercapillarisation and volumetric proportions of placental histological components stratified by fetal sex in term pregnancies; Supplementary Table S3. Effect of gestational age and infection inclusive of maternal BMI on placental maturity stratified by fetal sex; Supplementary Table S4. Placental morphometry volumetric proportions amongst preterm with chorioamnionitis and preterm without chorioamnionitis pregnancies stratified by fetal sex; Supplementary Table S5. Odds of placental pathology for preterm with chorioamnionitis pregnancies in comparison to preterm pregnancies without chorioamnionitis.
