# Supplementary material for: Gestational Age, Infection, and Suboptimal Maternal Prepregnancy BMI Independently Associate with Placental Histopathology in a Cohort of Pregnancies without Major Maternal Comorbidities
Source: J Clin Med. 2024 Jun 8;13(12):3378. doi: 10.3390/jcm13123378 (PMC11204067; doi:10.3390/jcm13123378)
Supplement: Supplementary file 1 [file jcm-13-03378-s001.zip › Supplementary Figures.pdf]

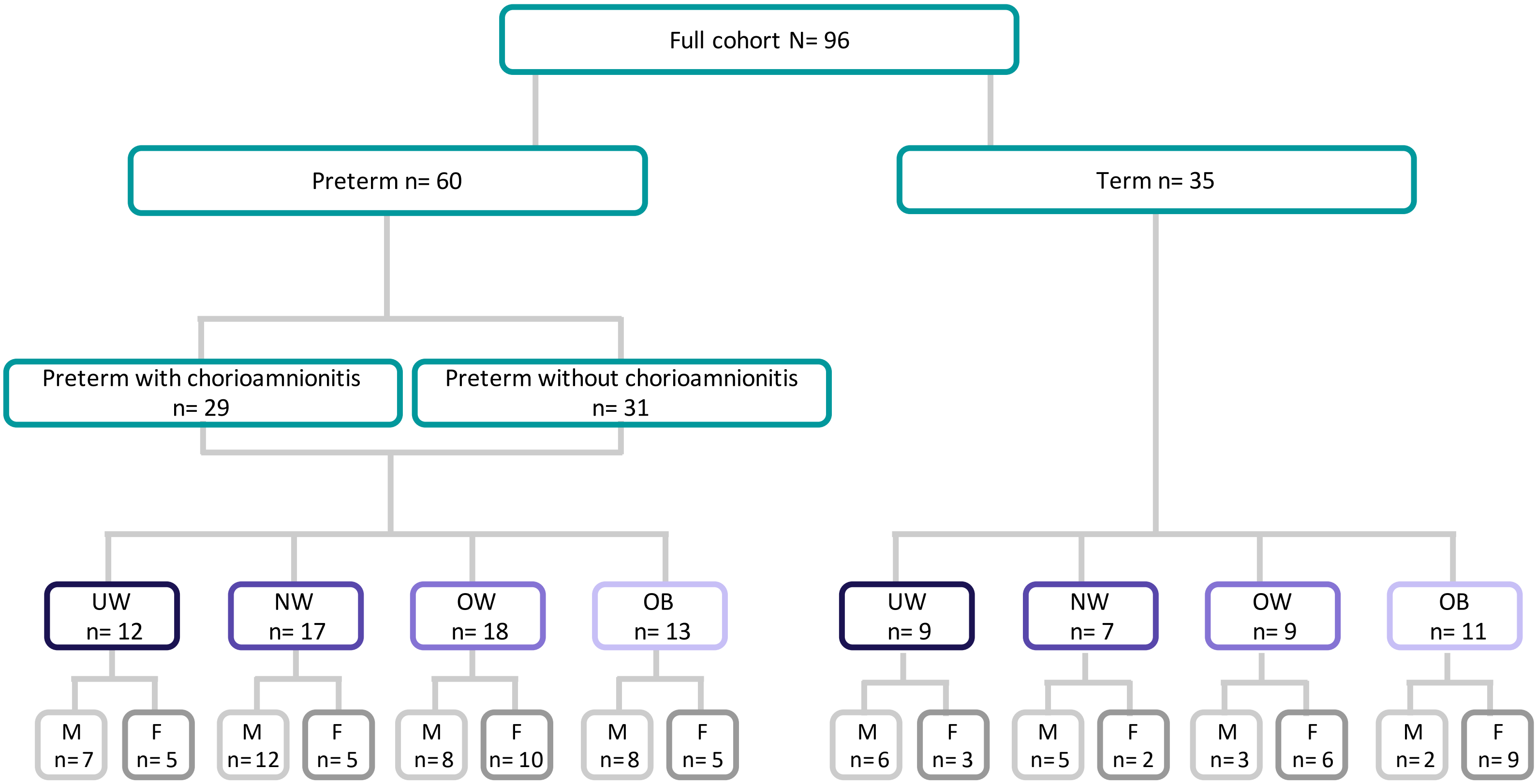

Supplementary Figure S1. Cohort breakdown

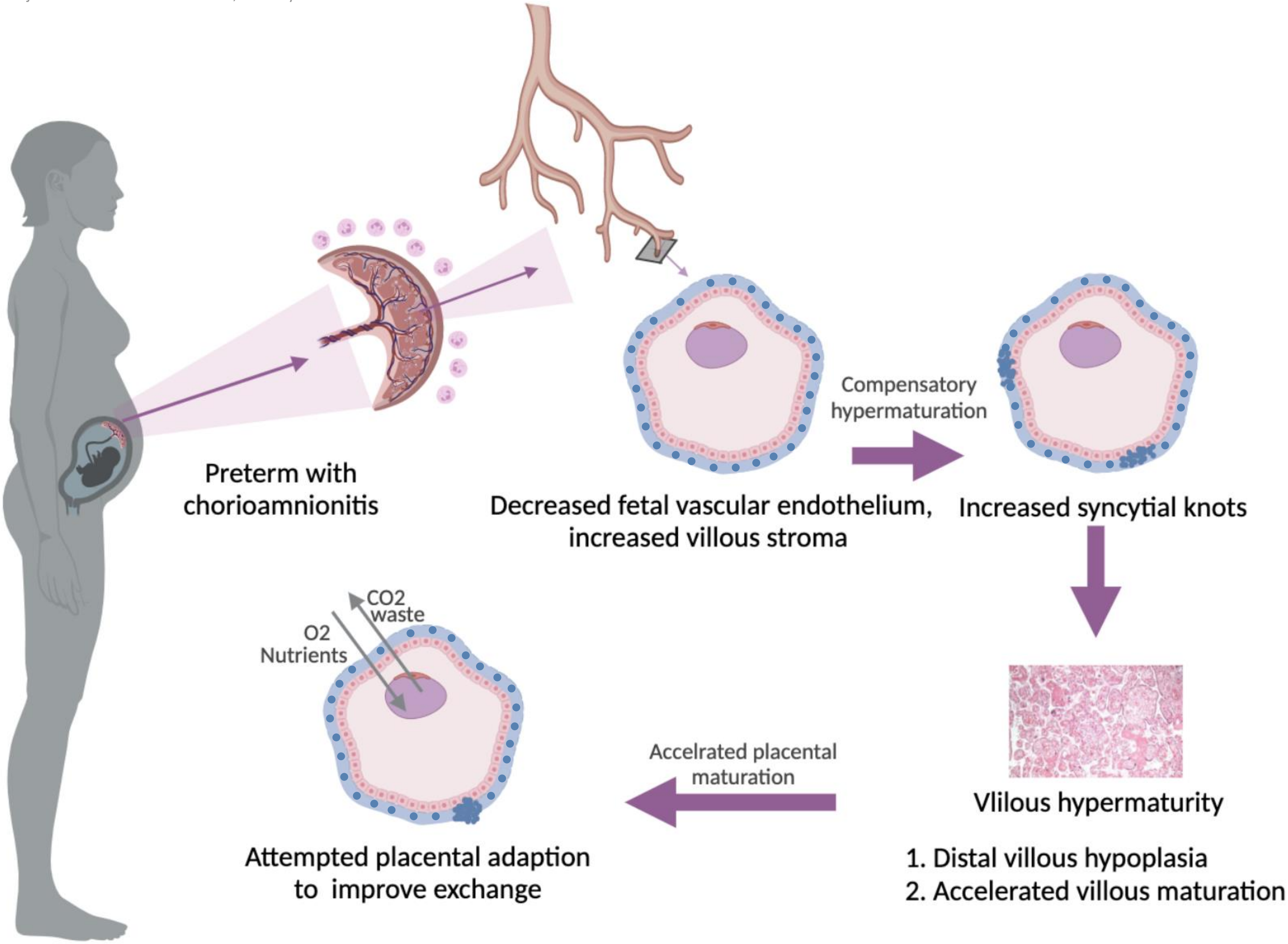

Supplementary Figure S2. Proposed mechanism of placental villous hypermaturity in preterm pregnancies with chorioamnionitis. Decreased fetal vascular endothelium and increased villous stroma, and subsequently inadequate placental vasculature and blood flow, in preterm pregnancies with chorioamnionitis may prompt compensatory placental hypermaturation. Syncytiotrophoblast shedding via increased number of syncytial knots and subsequent thinning of the syncytiotrophoblast layer may be one mechanism leading to placental villous hypermaturity and attempted improved exchange in preterm pregnancies with chorioamnionitis.
