# Supplementary material for: Gestational Age, Infection, and Suboptimal Maternal Prepregnancy BMI Independently Associate with Placental Histopathology in a Cohort of Pregnancies without Major Maternal Comorbidities
Source: J Clin Med. 2024 Jun 8;13(12):3378. doi: 10.3390/jcm13123378 (PMC11204067; doi:10.3390/jcm13123378)
Supplement: Supplementary file 1 [file jcm-13-03378-s001.zip › Supplementary Tables.pdf]

Supplementary Table S1. Effect of maternal BMI group on volumetric proportions of placental histological components and placental maturity stratified by fetal sex in preterm pregnancies.

	UW	NW	OW	OB	p value	q value
**Placental maturity (n (%))**						
**Male**	(n= 6)	(n= 12)	(n= 8)	(n= 8)	0.05	0.1
Immature	0 (0.00)	0 (0.00)	2 (25.0)	2 (25.0)		
Normal	6 (100)	6 (50.0)	6 (75.0)	4 (50.0)		
Hypermature	0 (0.00)	6 (50.0)	0 (0.00)	2 (25.0)		
**Female**	(n= 4)	(n= 5)	(n= 10)	(n= 4)	0.62	0.62
Immature	1 (25.0)	1 (20.0)	3 (30.0)	1 (25.0)		
Normal	1 (25.0)	3 (60.0)	4 (40.0)	3 (75.0)		
Hypermature	2 (50.0)	1 (20.0)	3 (30.0)	0 (0.0)		
**Placental morphometry: volumetric proportion of histological components (%)**						
**Male**	(n= 7)	(n= 11)	(n= 7)	(n= 9)		
Syncytiotrophoblast	20 (17.5, 20.5)	21 (19, 21.8)	20.5 (18.3, 23.8)	20 (19.3, 25)	0.69	0.91
Cytotrophoblast	0 (0, 0.25)	0 (0, 0)	0 (0, 0)	0 (0, 0)	0.39	0.78
Villous stroma	55.5 (54, 62)	57 (42.3, 62.3)	54.5 (47.3, 58.5)	53.5 (46.3, 61)	0.81	0.91
Fetal capillaries	22.8 ± 5.03	25.3 ± 11.5	24.3 ± 5.28	25.1 ± 9.85	0.95	0.95
Syncytial knots	1 (0, 1.25)	0 (0, 1)	1 (0, 2)	0.5 (0, 1)	0.57	0.91
**Female**	(n= 3)	(n= 5)	(n= 9)	(n= 5)		
Syncytiotrophoblast	22 (20, 23)	20 (18.5, 23.7)	22 (19.8, 25.5)	17 (10.8, 24.8)	0.25	0.78
Cytotrophoblast	0 (0, 0)	0 (0, 0.03)	0 (0, 0)	0 (0, 0)	0.35	0.78
Villous stroma	57 (53, 57)	62 (58.6, 64.5)	57.5 (53, 59.3)	53.5 (49.8, 55.8)	0.12	0.6
Fetal capillaries	21.3 ± 3.81	17.0 ± 2.95	19.7 ± 2.09	28.8 ± 3.30	0.09	0.6
Syncytial knots	1 (1, 2)	1 (0.53, 1.5)	1 (0, 2)	0.5 (0, 1.75)	0.82	0.91

Data are presented as means ± SD (ANOVA; normal distribution/equal variance), median (IQR; Kruskal-Wallis test for non-parametric data, or n (%) (Likelihood Ratio Chi Square test) where *p<0.05.

Supplementary Table S2. Effect of maternal BMI group on placental maturity, hypercapillarisation and volumetric proportions of placental histological components stratified by fetal sex in term pregnancies.

	UW	NW	OW	OB	p value	q value
**Placental maturity (n (%))**						
**Male**	(n= 6)	(n= 5)	(n= 2)	(n= 1)	0.41	0.41
Immature	2 (33.3)	1 (20.0)	1 (50.0)	1 (100)		
Normal	4 (66.7)	4 (80.0)	1 (50.0)	0 (0.00)		
Hypermature	0 (0.00)	0 (0.00)	0 (0.00)	0 (0.00)		
**Female**	(n= 2)	(n= 2)	(n= 6)	(n= 7)	0.31	0.41
Immature	1 (50.0)	0 (0.00)	4 (66.7)	4 (57.1)		
Normal	1 (50.0)	2 (100)	2 (33.3)	3 (42.9)		
Hypermature	0 (0.00)	0 (0.00)	0 (0.00)	0 (0.00)		
**Hypercapilarisation (n (%))**						
**Male**					0.09	0.24
Present	1 (16.7)	0 (0.00)	1 (50.0)	1 (100)		
Absent	5 (83.3)	5 (100)	1 (50.0)	0 (0.00)		
**Female**					0.12	0.24
Present	0 (0.00)	0 (0.00)	4 (66.7)	2 (28.6)		
Absent	2 (100)	2 (100)	2 (33.3)	5 (71.4)		
**Placental morphometry: volumetric proportion of histological components (%)**						
**Male**	(n= 6)	(n= 5)	(n= 2)	(n= 1)		
Syncytiotrophoblast	20.5 (17.7, 23.2)	23 (19, 23.6)	15.5 (15, 16)	17 (17, 17)	0.22	0.73
Cytotrophoblast	0 (0, 0.25)	0 (0, 0)	0 (0, 0)	0 (0, 0)	0.76	0.84
Villous stroma	51.5 (44.2, 55)	56 (50.5, 59.6)	55.5 (55, 56)	50 (50, 50)	0.40	0.8
Fetal capillaries	27.6 ± 3.19	23.0 ± 3.49	29.0 ± 5.52	32.0 ± 7.8	0.62	0.84
Syncytial knots	0.5 (0, 1.25)	0 (0, 0.8)	0 (0, 0)	0 (0, 0)	0.54	0.84
**Female**	(n= 2)	(n= 3)	(n= 5)	(n= 7)		
Syncytiotrophoblast	19 (18, 20)	19 (16, 22)	20 (15, 23)	20 (15, 22)	0.99	0.99
Cytotrophoblast	0 (0, 0)	0.5 (0, 1)	0 (0, 0.25)	0 (0, 0)	0.39	0.8
Villous stroma	55.5 (51, 60)	61.5 (60, 63)	46 (38.5, 58.8)	56 (55.5, 56)	0.13	0.65
Fetal capillaries	25.5 ± 4.95	19.0 ± 1.41	33.0 ± 9.63	24.6 ± 4.28	0.12	0.65
Syncytial knots	0.5 (0, 1)	0 (0, 0)	0 (0, 1)	0 (0, 1.5)	0.75	0.84

Data are presented as means ± SD (ANOVA; normal distribution/equal variance), median (IQR; Kruskal-Wallis test for non-parametric data), or n (%) (Likelihood Ratio Chi Square test) where *p<0.05.

Supplementary Table S3. Effect of gestational age and infection inclusive of maternal BMI on placental maturity stratified by fetal sex.

	**Preterm with chorioamnionitis**	**Preterm**	**Term**	**p value**	**q value**
**Placental maturity (n (%))**					
**Male**	(n= 15)	(n= 19)	(n= 14)	0.42	0.12
Immature	1 (6.67)	3 (15.8)	5 (35.7)		
Normal	9 (60.0)	13 (68.4)	9 (64.3)		
Hypermature	5 (33.3)	3 (15.8)	0 (0.00)		
**Female**	(n= 12)	(n= 10)	(n= 15)	0.06	0.42
Immature	0 (0.00)	5 (50.0)	9 (52.9)		
Normal	6 (50.0)	5 (50.0)	8 (47.1)		
Hypermature	6 (50.0)	0 (0.00)	0 (0.00)		

Data are presented as means ± SD (ANOVA; normal distribution/equal variance), median (IQR; Kruskal-Wallis test for non-parametric data), or n (%) (Likelihood Ratio Chi Square test) where *p<0.05.

Supplementary Table S4. Placental morphometry volumetric proportions amongst preterm with chorioamnionitis and preterm without chorioamnionitis pregnancies stratified by fetal sex.

Volumetric proportion of placental histological components (%)	Preterm with chorioamnionitis	Preterm without chorioamnionitis	p value	q value
**Male**	(n= 15)	(n= 19)		
Syncytiotrophoblast	20 (19, 22)	20 (19, 21)	0.55	0.78
Cytotrophoblast	0 (0, 0)	0 (0, 0)	0.86	0.86
Villous stroma	59 (51, 53)	55 (46, 59)	0.15	0.62
Fetal capillaries	22.8 ± 7.8	26.1 ± 9.3	0.28	0.62
Syncytial knots	1 (0, 1)	0 (0, 1)	0.83	0.86
**Female**	(n= 12)	(n= 10)		
Syncytiotrophoblast	20.5 (19.2, 23)	22 (19, 24.5)	0.67	0.67
Cytotrophoblast	0 (0, 0)	0 (0, 0)	0.31	0.46
Villous stroma	57.5 (55.5, 62)	55.5 (49.7, 59.2)	0.23	0.46
Fetal capillaries	19.6 ± 5.06	22.5 ± 9.39	0.37	0.46
Syncytial knots	1 (1, 2)	1 (0, 1)	0.11	0.46

Data are presented as means ± SD (ANOVA; normal distribution/equal variance with Tukey *post hoc*) or median (IQR; Kruskal-Wallis test for non-parametric data), where *p<0.05.

Supplementary Table S5. Odds of placental pathology for preterm with chorioamnionitis pregnancies in comparison to preterm pregnancies without chorioamnionitis

**Placental pathology**			**Odds of placental pathology in preterm with chorioamnionitis (vs preterm)**	
	Preterm with chorioamnionitis	Preterm	OR (95% CI)	aOR (95% CI)
**Accelerated villous maturation (AVM) (n [%])**	(n= 27)	(n= 29)		
Present	11 (40.7)	3 (10.3)	6.0 (0.04, 0.69)**	2.29 (0.36, 14.5)
**Distal villous hypoplasia (DVH) (n [%])**				
Present	5 (18.5)	1 (3.4)	6.4 (0.69, 58.1)	1.57 (0.07, 36.7)

Data are n (%) and odds ratios or adjusted odds ratios (95% CI) for presence of pathology in preterm with chorioamnionitis placentae (reference group: preterm) from Nominal Logistic regression models with p value from Likelihood Ratio Chi Square test. *p<0.05. Statistical significance is denoted as, **p=0.01. CI, confidence interval. OR, odds ratio. aOR, adjusted odds ratio. aOR adjusted for fetal sex, maternal gestational weight gain, maternal prepregnancy BMI and gestational age.
